# Supplementary material for: Spatial variation in allometric growth of invasive lionfish has management implications
Source: PeerJ. 2019 Apr 2;7:e6667. doi: 10.7717/peerj.6667 (PMC6450370; doi:10.7717/peerj.6667)
Supplement: Table S1 — Coordinates, minimum, maximum and mean depth (m), and number of samples for each location. [file peerj-07-6667-s001.pdf]

Spatial variation in allometric growth of invasive lionfish has management implications

Supplementary Table 1

Villaseñor-Derbez & Fitzgerald

Coordinates, minimum, maximum and mean depth (m), and number of samples for each location.

Location	Lat.	Long.	Min. Depth	Max. Depth	Mean Depth	n
Canones	20.477	-87.233	15.0	31.2	21.6	11
Castillo	20.496	-87.220	12.5	30.5	27.5	18
Cuevitas	20.478	-87.244	7.4	12.8	11.2	4
Islas	20.490	-87.228	14.0	19.4	16.7	10
Paamul	20.513	-87.192	9.9	22.7	15.5	31
Paraiso	20.484	-87.226	9.4	38.1	17.7	16
Pared	20.502	-87.212	12.1	21.0	16.3	12
Pedregal	20.507	-87.204	14.4	14.9	14.7	3
Santos	20.493	-87.222	5.7	26.6	16.2	2
Tzimin-Ha	20.393	-87.307	21.2	24.6	22.9	2
Total			5.7	38.1	18.6	109
