# Supplementary material for: Spatial variation in allometric growth of invasive lionfish has management implications
Source: PeerJ. 2019 Apr 2;7:e6667. doi: 10.7717/peerj.6667 (PMC6450370; doi:10.7717/peerj.6667)
Supplement: Figure S1 — Log-log transformation of the length-weight relationships ( n = 18) for 12 studies and this study. The curves are shown for the range of lengths reported in each study (See Table S2); when ranges were not present, we use the ones found in this study (34 mm–310 mm). Colors indicate studies from which the parameters were extracted. Dotted, dashed, and solid lines show models for males, females, and combined sexes, respectively. The dashed black line represents the relationship estimated in this study. There are two solid green lines for Sabido-Itza et al, 2016b, one for each of the two sites for which they report parameters. A linear-linear version of this figure is presented in Fig. 3 of the main text. [file peerj-07-6667-s003.pdf]

Spatial variation in allometric growth of invasive lionfish has management implications

Supplementary Figure 1

Villaseñor-Derbez & Fitzgerald

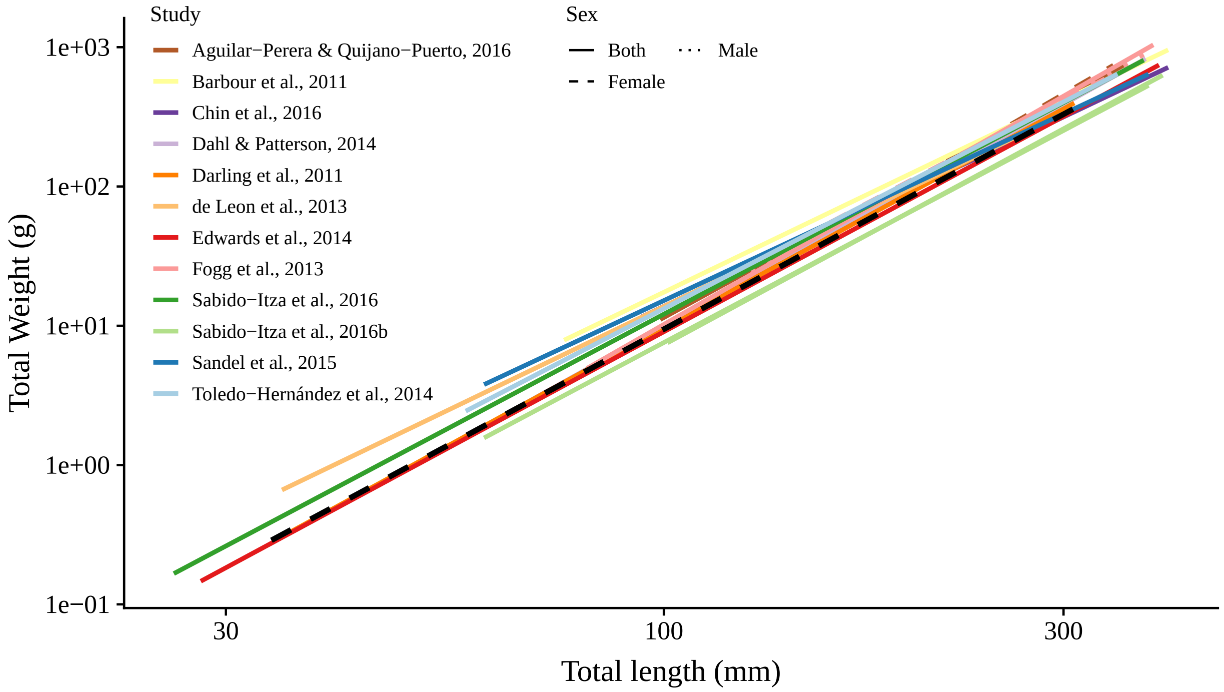

Figure 1: Log-log transformation of the length-weight relationships (n = 18) for 12 studies and this study. The curves are shown for the range of lengths reported in each study (See Supplementary Table 2); when ranges were not present, we use the ones found in this study (34 mm - 310 mm). Colors indicate studies from which the parameters were extracted. Dotted, dashed, and solid lines show models for males, females, and combined sexes, respectively. The dashed black line represents the relationship estimated in this study. There are two solid green lines for Sabido-Itza et al, 2016b, one for each of the two sites for which they report parameters. A linear-linear version of this figure is presented in Figure 3 of the main text.
