# Supplementary material for: Spatial variation in allometric growth of invasive lionfish has management implications
Source: PeerJ. 2019 Apr 2;7:e6667. doi: 10.7717/peerj.6667 (PMC6450370; doi:10.7717/peerj.6667)
Supplement: Table S2 — Summary of 18 allometric growth parameters available for lionfish in the invaded range from peer-reviewed literature and this study. All parameters have been adjusted to convert from millimeters to grams. n =Sample size, Sex specifies whether data was presented for Females (F), Males (M), or both genders combined (B), a, scaling parameter, b, exponent. Total Lenth ranges for Barbour et al. (2011) and Sandel et al. (2015) were estimated from figures since they did not explicitly report these parameters. An asterisk (*) in the Spear column indicates that at least some samples were obtained with pole spears. [file peerj-07-6667-s002.pdf]

Spatial variation in allometric growth of invasive lionfish has management implications

Supplementary Table 2

Villaseñor-Derbez & Fitzgerald

Extension of data from Table 1 in the main text. This table includes all the variables collected in each study.

Table 1: Summary of 18 allometric growth parameters available for lionfish in the invaded range from peer-reviewed literature and this study. All parameters have been adjusted to convert from millimeters to grams. n = Sample size, Sex specifies whether data was presented for Females (F), Males (M), or both genders combined (B), a = scaling parameter, b = exponent. Total Lenth ranges for Barbour et al (2011) and Sandel et al (2015) were estimated from figures since they did not explicitly report these parameters. An asterisk (*) in the Spear column indicates that at least some samples were obtained with pole spears.

Region	Sex	n	TL range (mm)	a	b	R^2	Depth range (m)	Years	Spear	Study
Western Atlantic	B	774	75 - 475	2.9	2.89	-	27 - 45	2004 - 2009	Yes*	Barbour et al., 2011
Western Atlantic	B	-	-	0.25	3.29	-	-	NA	NA	Darling et al., 2011
GoM	B	934	67 - 377	0.21	3.34	0.98	-	2013 - 2014	Yes	Dahl & Patterson, 2014
GoM	B	472	98 - 358	0.29	3.30	0.95	5 - 20	2011 - 2013	Yes	Aguilar-Perera & Quijano-Puerto, 2016
GoM	F	67	152 - 345	0.12	3.47	0.95	5 - 20	2011 - 2013	Yes	Aguilar-Perera & Quijano-Puerto, 2016
GoM	M	59	151 - 325	0.42	3.23	0.95	5 - 20	2011 - 2013	Yes	Aguilar-Perera & Quijano-Puerto, 2016
GoM	B	582	80 - 385	0.14	3.43	0.99	-	2012	Yes*	Fogg et al., 2013
GoM	M	119	126 - 385	0.27	3.31	0.97	-	2012	Yes*	Fogg et al., 2013
GoM	F	115	157 - 337	0.68	3.14	0.94	-	2012	Yes*	Fogg et al., 2013
Caribbean	B	458	60 - 380	3.6	2.81	-	-	2011	Yes*	Sandel et al., 2015
Caribbean	B	419	93 - 410	2.8	2.85	0.8715	18.3 - 18.3	2012 - 2014	Yes	Chin et al., 2016
Caribbean	B	1450	-	2.3	2.89	0.9216	-	-	-	de Leon et al., 2013
Caribbean	B	1887	27 - 391	0.3	3.24	0.97	15 - 30	2011	Yes*	Edwards et al., 2014
Caribbean	B	2143	25 - 375	0.52	3.18	0.9907	0.5 - 57	2009-2012	Yes*	Sabido-Itza et al., 2016
Caribbean	B	227	57.09 - 349	0.8	3.11	0.958	1 - 30	2010 - 2011	Yes*	Toledo-Hernández et al., 2014
Caribbean	B	449	100 - 395	0.23	3.25	0.97	-	2012-2013	Yes	Sabido-Itza et al., 2016b
Caribbean	B	368	60 - 380	0.32	3.19	0.98	-	2012-2013	Yes	Sabido-Itza et al., 2016b
Caribbean	B	109	34 - 310	0.32	3.23	0.9766	5.7 - 38.1	2010	No	This study
